# Raw Data Visualization for Common Factorial Designs Using SPSS: A Syntax Collection and Tutorial

**DOI:** 10.3389/fpsyg.2022.808469

**Published:** 2022-03-30

**Authors:** Florian Loffing

**Affiliations:** ^1^Institute of Sport Science, Carl von Ossietzky University Oldenburg, Oldenburg, Germany; ^2^Institute of Psychology, German Sport University, Cologne, Germany

**Keywords:** univariate distribution, descriptive, continuous data, teaching, statistics, quantitative methods

## Abstract

Transparency in data visualization is an essential ingredient for scientific communication. The traditional approach of visualizing continuous quantitative data solely in the form of summary statistics (i.e., measures of central tendency and dispersion) has repeatedly been criticized for not revealing the underlying raw data distribution. Remarkably, however, systematic and easy-to-use solutions for raw data visualization using the most commonly reported statistical software package for data analysis, IBM SPSS Statistics, are missing. Here, a comprehensive collection of more than 100 SPSS syntax files and an SPSS dataset template is presented and made freely available that allow the creation of transparent graphs for one-sample designs, for one- and two-factorial between-subject designs, for selected one- and two-factorial within-subject designs as well as for selected two-factorial mixed designs and, with some creativity, even beyond (e.g., three-factorial mixed-designs). Depending on graph type (e.g., pure dot plot, box plot, and line plot), raw data can be displayed along with standard measures of central tendency (arithmetic mean and median) and dispersion (95% CI and SD). The free-to-use syntax can also be modified to match with individual needs. A variety of example applications of syntax are illustrated in a tutorial-like fashion along with fictitious datasets accompanying this contribution. The syntax collection is hoped to provide researchers, students, teachers, and others working with SPSS a valuable tool to move towards more transparency in data visualization.

## Introduction

Data visualization is an important means to communicate scientific results (Anscombe, 1973; Tufte, 2001; Duke et al., 2015; Taamneh et al., 2016). Continuous quantitative data are often visualized in the form of summary statistics, with a measure of central tendency (e.g., arithmetic mean) being displayed together with a measure of dispersion (e.g., SD, CI). Dispersion measures are considered as an integral part of the visualization of continuous data to indicate, in the case of SD, the “average” variation of individual data points around the observed arithmetic mean or to indicate, in the case of standard error of the mean (SEM) and CI, the precision in the estimation of an unknown population parameter reflected in the observed arithmetic mean. However, dispersion measures are of limited value because they do not reveal the actual raw data distribution underlying a measure of central tendency. For example, they do neither conceal clearly whether the raw data follow a symmetric and unimodal distribution nor whether they include outliers (Weissgerber et al., 2015, 2019).

For small sample studies (i.e., up to 20–30 participants), which are sometimes inevitable in sports science research when, for example, considering elite athletes as participants, visualization of raw data is recommended over dispersion measures to better indicate inter-individual variation (Weissgerber et al., 2019). In the case of repeated-measures designs, visualization of only summary statistics hides whether individuals who score high (low) in one condition also score high (low) in another condition (e.g., aerobic running capacity with vs. without preferred music) or whether changes seen at the group level such as a pre-to-post improvement in executive functioning following an acute bout of exercise are consistently present also at the individual level. Revealing inter-individual consistency in performance change, however, may help strengthen a phenomenon’s underlying theory and would particularly constitute an easy-to-communicate strategy to visualize the potential practical utility of a treatment through highlighting (the proportion of) responders and non-responders (Nimphius and Jordan, 2020). The latter cannot properly be inferred from standardized effect sizes such as Cohen’s *d* (Cohen, 1988) because, even though they are recommended to be reported as quantitative indicators of practical significance, they relate to effects at the group level and not at the individual level (Ferger and Büsch, 2018; Turner et al., 2021).

Acknowledging the formerly mentioned limitations of data visualization, various editorials (Drummond and Vowler, 2011; Fosang and Colbran, 2015; Teare, 2016; Nimphius and Jordan, 2020) and journal articles (Weissgerber et al., 2015, 2017, 2019; Hertel, 2018) highlighted the need to improve transparency in data visualization through the presentation of raw data in addition to or even as substitute for classically reported summary statistics. Consequently, technical solutions have been provided recently (see Table 3 in Weissgerber et al., 2019, for an overview) to facilitate transparency in the presentation of univariate data such as Microsoft Excel templates for independent and paired samples (Weissgerber et al., 2015), tutorials for data visualization using GraphPad PRISM (Weissgerber et al., 2015) or a variety of open-accessible web-based applications (e.g., Weissgerber et al., 2016^1^; Mauri et al., 2017^2^; Ho et al., 2019^3^; Postma and Goedhart, 2019^4^). Also, popular open-source statistic programs like JASP (JASP Team, 2022) or JAMOVI (The Jamovi Project, 2021) allow the addition of raw data to certain summary graphs such as box plots.

Strikingly, IBM SPSS Statistics, which is the most commonly reported statistical software package used for data analysis in scientific journal articles since more than two decades (Muenchen, 2013, 2019), does not provide the user-friendly menu-driven feature of raw data visualization along with measures of central tendency and dispersion until now. This may be one reason why transparency in data visualization has yet to become standard in scientific publishing despite the above cited advances in data presentation techniques (Weissgerber et al., 2019). Obviously, the absence of a directly accessible raw data visualization feature in SPSS complicates the creation of raw data plots since data would need to be transferred to another program or web-app to create the graphical output of interest. Closer inspection of the features that SPSS includes for graphical presentation, however, reveals that transparent data visualization indeed is possible, but only *via* syntax that relies on the Graphics Programming Language (GPL; Wilkinson, 2005). Savic (2016), as referred to in Weissgerber et al. (2019), provides instructions for the creation of univariate dot plots along with the median or arithmetic mean in the case of two or more independent groups. Apart from these instructions and some scattered online-discussions on related issues, for example, on ResearchGate (Preziosa, 2018), to date the cases covered by available SPSS syntax are limited and there appears to be no systematic collection of syntax solutions for transparent raw data visualization using SPSS. Likewise, that topic has not been addressed in detail up until the most recent edition of the possibly most popular SPSS textbook (Field, 2018) or other contributions more directly related to data visualization in SPSS (Aldrich and Rodríguez, 2013; McCormick et al., 2017).

The aim of this work is to counter the paucity of practical solutions available for transparent data visualization using IBM SPSS Statistics. A free-to-use collection of more than 100 syntax files is presented to encourage and to facilitate users of SPSS to create transparent visualizations of summary statistics and its underlying raw data.

## The SPSS Syntax Collection

### Preliminary Notes

The syntax collection has been created on a “Windows 10” operating system under IBM SPSS version 27, the code has been written in the SPSS-built-in language GPL (Wilkinson, 2005) and the code’s functionality has been tested on two operating systems (Windows 10, macOS Big Sur version 11.5.2) under the default chart settings in SPSS (“Edit > Options… > Charts > Chart Template > Use Current Settings”). Note that when using non-default chart settings (e.g., installed chart templates such as APA) the syntax-based graphical outputs will differ and occasionally might turn out inappropriate for further use.

Users familiar with SPSS know that, by default, the syntax code that is run is also shown in the SPSS Viewer window on top of a graphical output. Since this makes the output unnecessarily long, users may not want this to happen. To hide an output’s underlying syntax in the viewer from the outset, simply tell SPSS to do so (“Edit > Options … > Viewer > Initial Output State > Item: Log > Contents are initially: Hidden”). Note that setting the log-status to “hidden” has effect on all future outputs such that syntax code is always hidden for any process run and not only for the syntax provided with this article. However, code is not lost and can still be made visible afterwards, for example, by double-clicking on the Log-symbol in the left column of the SPSS Viewer window.

### Study Designs and Graph Types Covered

A summary of study designs, examples of corresponding parametric tests and the graph types available for each design through syntax is given in Table 1. The syntax collection covers common, basic study designs such as one-sample design, one- and two-factorial between-subject designs, one- and two-factorial within-subject designs as well as two-factorial mixed designs.

**Table 1:** Overview of study designs and graph types covered by the SPSS-syntax collection.

Design	Grouping variables in SPSS	Measurement variables in SPSS	Examples of parametric statistical tests	Pure dot plot	Box plot with dots	Line plot with dots	Bar plot with dots
One-sample	0	1	One-sample *t*-test	✓	✓	×	×
One between-subject factor	1 (≥2 levels)	1	*t*-Test for independent samples, one-way univariate ANOVA	✓	✓	×	✓
Two between-subject factors	2 (IV_bs-1_ and IV_bs-2_: ≥2 levels)	1	Two-factorial univariate ANOVA	✓	✓	×	✓
One within-subject factor	0	2	*t*-Test for dependent samples	✓^Δ^	✓^a^^,^ ^Δ^	✓^a^^,^ ^Δ^	×
		3	One-way repeated-measures ANOVA (e.g., pre-post-retention)	✓^Δ^	✓^a^^,^ ^Δ^	✓^a^^,^ ^Δ^	×
		4 or 5	One-way repeated-measures ANOVA	✓	✓^a^	✓^a^	×
Mixed design (1 between-subject factor x 1 within-subject factor)	1 (IV_bs_: 2 levels)	2 (IV_ws_)	Two-factorial mixed ANOVA with repeated measures on the within-subject factor	✓^Δ^	✓^a^^,^ ^Δ^	✓^a^^,^ ^Δ^	×
		3 (IV_ws_)		✓^Δ^	✓^a^^,^ ^Δ^	✓^a^^,^ ^Δ^	×
		4 or 5 (IV_ws_)		✓	✓^a^	✓^a^	×
	1 (IV_bs_: 3 levels)	2 (IV_ws_)		✓^Δ^	✓^a^^,^ ^Δ^	✓^a^^,^ ^Δ^	×
		3 (IV_ws_)		✓^Δ^	✓^a^^,^ ^Δ^	✓^a^^,^ ^Δ^	×
		4 or 5 (IV_ws_)		✓	✓^a^	✓^a^	×
Two within-subject factors	0	4 (IV_ws-A_ [2 levels] x IV_ws-B_ [2 levels])	Two-factorial repeated measures ANOVA	✓ (difference scores only)	×	×	×
		6 (IV_ws-A_ [2 levels] x IV_ws-B_ [3 levels])		✓ (difference scores only)	×	×	×

a Two visualization options are available: Individual dots (i.e., cases in a dataset) connected by grey solid lines (filenames include the string “raw-dots-CONNECTED”) or not connected (filenames include the string “raw-dots-NOT-connected”; see main text for details).

Δ An additional panel with difference score(s) can optionally be created as well. The corresponding syntax filenames include the string “with-Delta.”

IV = independent variable. In the case of two-factorial designs, subscripts differentiate between the two independent variables (bs = between-subject factor, 1 = factor 1, 2 = factor 2; ws = within-subject factor, A = factor A, B = factor B).

For one- or two-factorial between-subject designs, syntax is not restricted to the number of factor levels or its combinations. For one-factorial within-subject designs, data from up to five levels of a within-subject factor can be visualized. Similarly, for mixed designs graphs showing two to five levels of the within-subject factor in combination with two or three levels of the between-subject factor can be created. Moreover, for one-factorial within-subject designs and mixed designs, two versions of syntax are available that lead to very similar graphs. The key difference between versions is that the dots representing raw data are either connected by straight lines (syntax filenames include “raw-dots-CONNECTED”) between adjacent levels of the within-subject factor or not (syntax filenames include “raw-dots-NOT-connected”). Connecting raw dots is recommended only when a within-subject factor has two levels or when the levels of the within-subject factor follow a clear, pre-specified order (e.g., temporal order like pre-test, post-test, and retention-test). Otherwise, connecting individual dots by lines can be misleading since the ordering of within-subject levels may not be clearly defined. In the latter case, it is recommended to create graphs without connecting the raw data dots. Due to the enhanced complexity and relatedness of data in pure two-factorial within-subject designs, the code provided in the syntax collection is limited to 2-x-2 and 2-x-3 within-subject designs (see Two-Factorial Within-Subject Design below for details). For study designs including one within-subject factor with two or three levels, in addition to the observed data, difference scores between adjacent within-subject levels can also be visualized in separate panels.

The syntax collection allows the creation of commonly used graph types appropriate for the formerly mentioned study designs. Specifically, depending on study design, pure dot plots, box plots, line graphs and bar graphs with dots representing the raw data can be created. Graph types were selected for inclusion based on recent recommendations by Weissgerber et al. (2019), with the only exception being the additional inclusion of syntax for bar graphs in between-subject designs. Note that syntax for bar graphs is made available in the collection mostly for nostalgic reasons as its use is not recommended for the visualization of continuous quantitative data (see, e.g., Weissgerber et al., 2017, for an in-depth discussion).

The syntax files are provided separately for study design and graph type. Moreover, each syntax file includes code on more than one graphical output. Specifically, for pure dot plots, line and bar graphs, users can choose between seven options for the display of measures of central tendency (arithmetic mean and median) and dispersion (SD, 95% CI) together with raw data (see Table 2). Box plots, in turn, may be created in its standard format without the arithmetic mean or in an extended format that includes the mean displayed as red horizontal line by default.

**Table 2:** Options for the visualization of measures of central tendency and dispersion, in addition to raw data, depending on graph type.

Measures	Dot plot	Box plot	Line plot	Bar plot
Mean(s) + 95% CI	✓	×	✓	✓
Mean(s) + SD	✓	×	✓	✓
Mean(s)	✓	✓	✓	✓
Median(s)	✓	×	✓	✓
Mean(s) + median(s)	✓	×	✓	✓
Mean(s) + 95% CI + median(s)	✓	×	✓	✓
Mean(s) + SD + median(s)	✓	×	✓	✓

The order of measures listed from the table’s top to bottom corresponds to the order of code included in the respective syntax files. By default, in all graphs that can be created with the SPSS-syntax templates mean values are indicated by red horizontal lines (except for bar graphs) and median values are indicated by blue horizontal lines. CI = confidence interval, SD = standard deviation. Note that CIs are visualized as stand-alone intervals that are related to their respective arithmetic means. Therefore, inference of between- or within-subject comparisons from visual inspection of CIs is not permitted (see Cousineau et al., 2021, for an R-based solution to that problem).

The syntax files are named so as to facilitate finding the file that suits a user’s need. Specifically, filenames provide information on (1) study design, (2) number of factors included in a design (one or two; does not apply to one-sample designs), (3) number of levels or combination of factor levels (applies to within-subject and mixed designs only), (4) graph type (dot, box, line, and bar), (5) whether dots representing raw data are connected or not and (6) whether graphs on difference scores calculated from repeated measures can be created as well (“with-Delta”; points 5–6 apply to within-subject and mixed designs only). All syntax files included in the collection are listed in Supplementary Table S1.

### SPSS Dataset Template

An SPSS dataset template (*SPSS_DataViz_Template.sav*) is provided along with the syntax collection. Please note that all syntax is coded with reference to the variables included in the dataset template. Therefore, it is recommended to work with the dataset template and to fill the variables needed for the creation of graphics. The dataset template can, of course, be extended with other variables collected as part of a study such as data on questionnaire items or the like. Conversely, the syntax collection could also be applied to other SPSS datasets a user works with; however, the code would then need to be adapted to the variable names used in that particular dataset.

**Figure 1:**
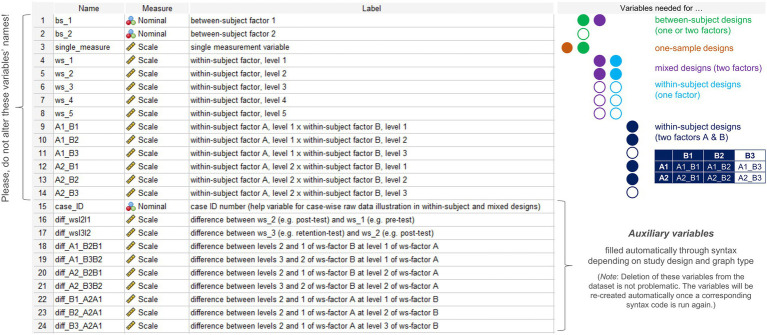
Sections taken from the variable view of the SPSS dataset template and variables needed to specify in the dataset depending on the study design or the factors/factor combination selected for data visualization. Color-filled circles denote essential variables for a given design, whereas white-filled color-bordered circles denote variables that are optional for a given design depending on the number of factors (one- or two-factorial between-subject designs), the number of within-subject factor levels in one-factorial within-subject or mixed designs (2–5 levels supported) or the number of levels of the second factor B (2 or 3 levels supported) in two-factorial within-subject designs.

The dataset template comprises 24 pre-specified variables (Figure 1). Variables 1–14 need to be filled with values depending on a user’s need (see section Example Applications). These variables should neither be deleted from the template nor their names be altered because then the syntax code would no longer function or the code would need to be rewritten to match with the altered variable names. Variables 15–24 are auxiliary variables that are filled automatically through syntax depending on the study design and graph type selected for graphical output. While these variables are already included in the dataset template, deletion of these variables will not impair syntax functioning. Instead, once a syntax code that relies on one or more auxiliary variables is run again, these variables will be re-created and added to the dataset template.

### Basic Workflow

**Figure 2:**
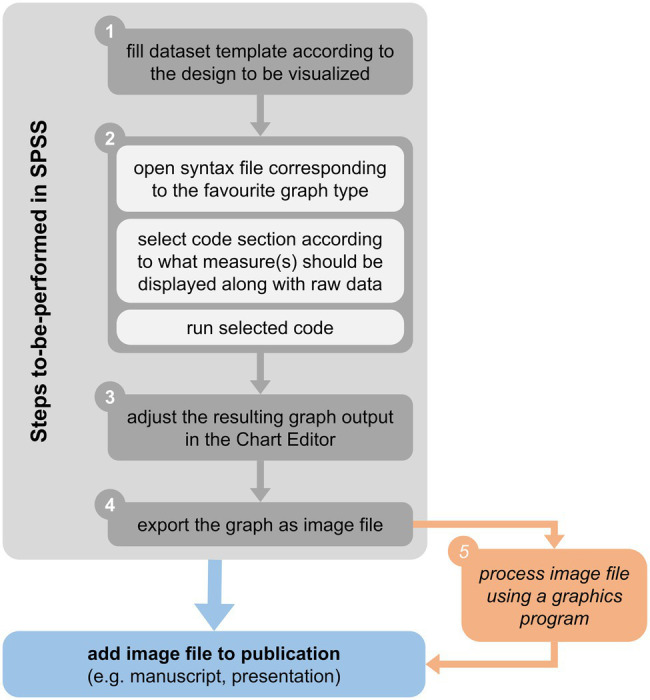
Basic workflow for raw data visualization in SPSS using syntax and the dataset template provided alongside this article (see main text for details).

The basic workflow underlying the syntax-based creation of graphics is illustrated in Figure 2 and described below with reference to the SPSS dataset template provided alongside this article. Concrete example applications of syntax on randomly sampled (i.e., fictitious) data will be described later in section Example Applications.

#### Step 1: Feed the Dataset Template With Data

In a first step, users need to type in or to paste data to variables 1–14 (see Figure 1) given the study design or its components one wishes to visualize (i.e., main effect or interaction) to have everything ready to create the graph(s) needed. Before proceeding with Step 2, however, in the variable view of SPSS users may additionally want to do the following optional settings: (a) if the intended visualization includes one or two grouping factors (i.e., variables “bs_1” and “bs_2”), assign values to the factor levels and (b) consider to assign meaningful, short labels to the variables one wishes to visualize. These optional steps allow to display factor levels and labels that are specific to the design in the graph from the outset.

Please note that for the visualization of data from two-factorial mixed designs, the to-be-displayed levels of the between-subject factor (stored in variable “bs_1”) need to be coded as 1 and 2 (in the case of a 2-levels between-subject factor) or as 1, 2, and 3 (in the case of a 3-level between-subject factor). Using other values for the coding of factor levels makes the corresponding syntax unusable for mixed-designs.

#### Step 2: Create the Graph Needed

Once all variables relevant to a particular study design are filled with values, the graph can be created. To do so, users need to choose the corresponding syntax file from the collection (see Supplementary Table S1 for a list of syntax files), open it and select the code segment that corresponds to the measures of central tendency and dispersion one wishes to display in the graph along with raw data (Table 2). To create the graph, in the SPSS syntax menu click “Run” and choose “Selection” (or use the shortcut Ctrl+R).

#### Step 3: Adjust the Graph Using the Chart Editor in SPSS

Following the previous step, the graph is created in a new output window. The graph’s layout and style are very basic such that further editing is highly recommended prior to inclusion in a manuscript or presentation. To do so, double-click on the graph such that a new window opens (Chart Editor). From there on, users can adjust, among others, chart size (with or without maintaining aspect ratio), font type, style and size of axis labels and ticks, range (e.g., minimum and maximum), and number format (e.g., decimal places) of values shown along the vertical axis as well as the type of scale (set to “linear” by default), marker color, type, and size for raw data points and for the chosen measure(s) of central tendency. Note that, when displaying CIs, the level set and visualized (95% by default) cannot be changed afterward in the Chart Editor. If users wish to visualize another level of confidence, they would need to modify syntax accordingly before running code (see section Modification of Syntax below for details).

#### Step 4: Export the Figure as Image

Once finished with Step 3, right-click on the image with the mouse-cursor and then choose “Export…” to open a new window called “Export Output.” Here, users need to ensure that in the window’s respective fields settings are made appropriately such that “Objects to Export > Selected” and “Document > Type: None (Graphics only).” To create an image that can be used at reasonable high spatial resolution in a later publication, it is recommended to further choose either “Graphics > Type: Production Ready Postscript (*.eps)” (or “Encapsulated Postscript (*.eps)” in former versions of SPSS) or “Graphics > Type: Portable Document Format (*.pdf).” The advantage of exporting graphics as pdf-file is that transparency settings for, e.g., raw data dots are maintained, while I found that this feature was lost and could not be restored when choosing the eps-option. Other common image file formats that might suit users’ needs are available as well such as *.emf, *.jpg and *.tif. Finally, in the “Root File Name” section at the bottom of the window set the path and file name for the image and click the “OK” button.

#### Step 5 (Optional): Image Processing Outside SPSS

Optionally, once Step 4 is completed the exported image might be processed further in another graphics program outside SPSS for finishing. This may be necessary, for example, if users wish to create a composite figure that consists of various single images each created with the former steps. In the example application sections that follow, each SPSS graphical output was exported as pdf-file, then that file was imported to the free graphical software GIMP (GIMP, 2021; options set during import: open pages as = layers, resolution = 300 pixels/inch, use anti-aliasing checked) and directly exported again (i.e., without any image processing steps in-between) as .tif-file [compression set to “none” and all other options checked (e.g. save layers, save color values from transparent pixels)]. The resulting tif-files were then used to create the composite figures shown in the sections below.

## Example Applications

In the following sections example applications of the syntax collection are illustrated separately for different types of study design. Readers may want to refer to these examples to learn more about the syntax and/or consider including it in teaching lessons. The respective examples’ underlying datasets are provided along with this article so as to facilitate replicating the following steps. Note that the examples do not cover all visualization options for all graph types but only provide a small selection of graphical outputs that can be created from syntax.

### Between-Subject Designs

Two example datasets are provided for pure between-subject designs, one to illustrate a one-factorial design with two levels (*dataset_example_BS-design_1-factor.sav*; fictitious expertise study with some measure of accuracy stored in the dataset variable “single_measure”; *cf.* Figure 1) and the other to illustrate a 2-x-2 design (*dataset_example_BS-design_2-factors.sav*; similar design as before but with the additional grouping factor “age”). Note that, theoretically, the syntax for pure between-subject designs is not limited to a particular number of factor levels or its combination in the case of two-factorial designs but should still be kept to a reasonable amount so as to not make visualizations difficult to read.

#### One-Factorial Between-Subject Design

**Figure 3:**
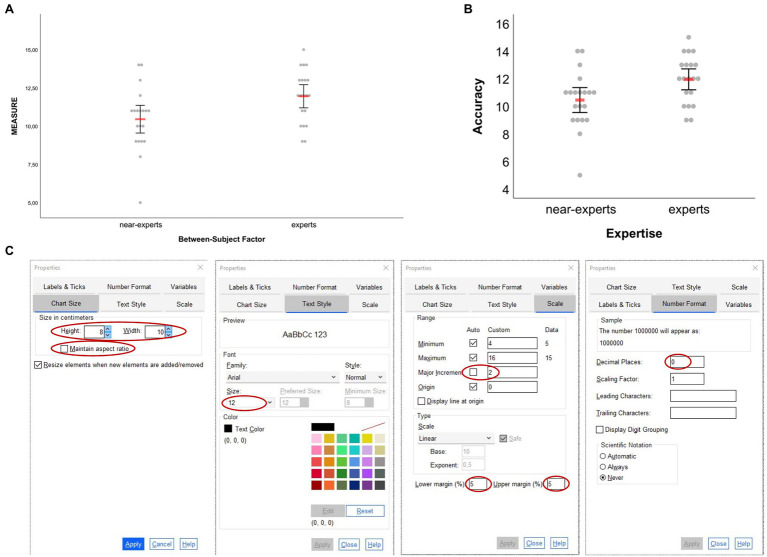
Dot plots created based on the example dataset for one-factorial between-subject designs. **(A)** Original output based on syntax and **(B)** adjusted figure based on the properties changes illustrated in **(C)**. In **(A**,**B)** the red horizontal bars represent the arithmetic mean and error bars represent 95% CIs associated with respective means. In **(C)**, the adjustments made are highlighted red (see main text for details).

The example visualization created for the one-factorial case shows a dot plot in its original form as obtained from the syntax file *BS-Design_1-factor_DOT-plot.sps* and the first visualization option provided therein to visualize arithmetic means with 95% CIs (Figure 3A; Step 2 in the basic workflow), its adjusted form (Figure 3B; Step 3 in basic workflow) and the adjustments made using the Chart Editor (Figure 3C). The same adjustments could also be applied to the other graph types available for one-factorial between-subject designs (i.e., bar and box plots) resulting in highly similar visualization layouts. If, in the dataset, no categorical values had been assigned to the different levels of the grouping factor “bs_1” as is done in the example dataset (i.e., 1 = near-experts, 2 = experts), then the numbers representing the different groups would have been displayed along the horizontal axis instead of categorical values. This is not problematic since by clicking twice on a particular category on the horizontal axis its value may still be edited and changed, for example, to “expert” in the case of level 2. By default, the horizontal axis is labelled as “Between-Subject Factor” and the vertical axis is labelled as “MEASURE.” Following the creation of a graphic using the syntax, these labels can be changed easily by also clicking twice on a label and then typing the label needed (in the example here: “Expertise” and “Accuracy”).

As can be inferred from Figure 3C, only a few settings were necessary to obtain the graph shown in Figure 3B. Once in the Chart Editor, double-clicking on the vertical axis opens a new window called “Properties” from where one can do all the settings illustrated in Figure 3C: chart size was reduced and before clicking “Apply” the “Maintain aspect ratio” button was unchecked, font size for numerical values along the vertical axis was set to 12 pt (note: chose value under “Size,” not “Preferred Size”), the vertical scale was adjusted by changing the major increment to 2, lower and upper margins were both set to 5% (this results in some distance of minimum and maximum values from horizontal axis and top of the figure) and the number of decimal places was set to 0 (set to 2 by default in this case). Not more than these few steps were made to modify the original output (Figure 3A) to its final layout (Figure 3B). Importantly, always click “Apply” before proceeding from one of the former property steps to the other; otherwise former settings may get lost and not apply to the graph. Note also that the property steps mentioned and illustrated in Figure 3C do not need to be conducted in that particular order, but can be realized in any order resulting in the same display. Likewise, the route to the “Properties” window mentioned above is not the only way to change a graphic’s properties. Alternatively, once in the Chart Editor, users could also do a right-click on the graph and select “Properties Window” from there on or simply use the shortcut “CTRL + T.” Of note, the property window that appears then likely differs from the one shown in Figure 3C and thus may allow fewer options for change (because the vertical axis was not selected first as illustrated above), but one can change options easily by then just clicking once on the particular graphical element that one would like to change in more detail (e.g., vertical or horizontal axis). In this regard, of course even more than the formerly described steps could have been applied to the graph, if needed, such as changing the color of raw dots, marker size, font size, or color for ticks or labels on the horizontal axis etc. Users irrespective of their skill in creating and editing graphs using SPSS will find out quickly how to realize the different types of changes to accommodate data visualizations to their individual aesthetic preference and/or to journal guidelines.

#### Two-Factorial Between-Subject Design

**Figure 4:**
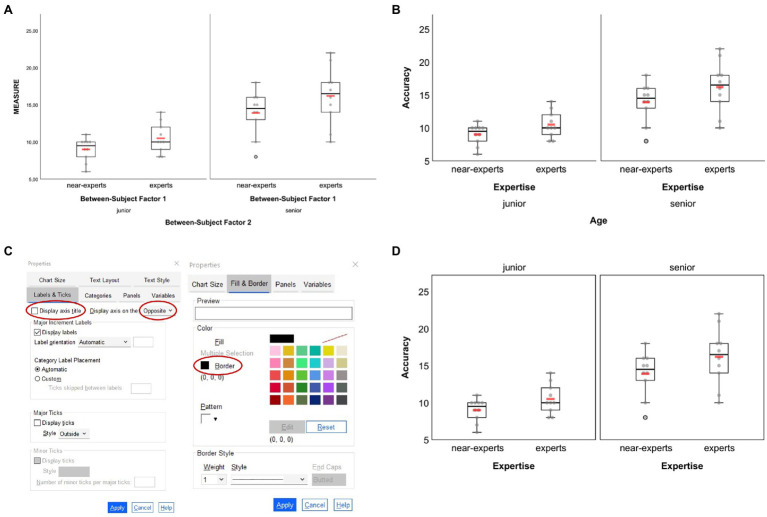
Box plots created based on the example dataset for two-factorial between-subject designs. **(A)** Original output based on syntax and **(B)** adjusted figure based on similar settings as for the one-factorial between-subject example. Further adjustment of properties related to the bottom horizontal axis (here: the second grouping factor “age”) as illustrated in **(C)** results in the figure displayed in **(D)**. In **(A**,**B**,**D)** each group’s arithmetic mean is represented through the red horizontal bars. In **(C)**, the adjustments made are circled red (see main text for details).

The two-factorial design example is similar to the former example but includes a second between-subject factor stored in the dataset variable “bs_2” that additionally differentiates between junior (1) and senior (2) experts and near-experts. As before, the dependent variable relates to some fictitious accuracy variable stored in the dataset variable called “single_measure” (cf. Figure 1). The box plot graphic shown in Figure 4A was created based on the syntax file *BS-Design_2-factors_BOX-plot.sps* and the second visualization option provided therein to create a standard box plot together with superimposed arithmetic means. As can be seen from Figure 4A, by default the graph is split into two panels according to the number of levels of the second between-subject factor “bs_2” (if that factor had, e.g., three levels, three panels would have been shown), while in each panel data are shown separately for each level of the first between-subject factor “bs_1.” Adjustments of the initial graphical output were realized in the Chart Editor *via* similar property changes as in the example before [one exception: for the vertical scale, default values for lower margin (0%) and upper margin (10%) were kept], which resulted in the graph shown in Figure 4B. Note that changing property values for the leftmost vertical scale (e.g., minimum, maximum, major increment, or margins) automatically applies to all panels. The second vertical axis that is located between both panels in the example illustration cannot be edited independently.

By default, horizontal axis titles and ticks are placed one below the other, resulting in four lines of text below a graph (see Figures 4A,B). Rather than having the labels for the second grouping factor in lines three and four, one can change their appearance by placing them above the respective panels. To do so, when still in the Chart Editor double-click on the fourth line (here: “Age”) and choose “Labels & Ticks” in the Properties window. Then, choose “Opposite” to display that particular axis above the panels. If the axis title (here: “Age”) should not be shown but only the two panels’ labels (here: “junior” and “senior”) then also uncheck the “Display axis title” option as shown in Figure 4C. As reminder, note that the vertical line of the second panel (here: “senior”) cannot be edited independently from the vertical axis of the first panel (here: “junior”). If there should not be a single vertical line between both panels, one solution could be to draw borders around the panels’ inner frame. To do so, click on one of the panels, press CTRL + T to open the Properties window and make the appropriate settings as shown in the right column of Figure 4C. The graph finally resulting from the former settings is given in Figure 4D. Of note, raw data dots that are considered as outliers are black-bordered as is shown for one senior near-expert participant in Figures 4A,B,D.

### Two-Factorial Mixed-Designs

For mixed-designs, remember that syntax allows to create graphs showing two to five levels of the within-subject factor in combination with two or three levels of the between-subject factor. With regard to the between-subject factor, it is important to keep in mind that the syntax only works with groups being coded as 1 and 2 (2-levels factor) or as 1, 2, and 3 (3-levels factor). Such restriction on factor-level coding does not apply to the pure between-subject designs formerly illustrated in section Between-Subject Designs.

The mixed-design example illustrated in the following relates to a fictitious training study with two groups (experimental, control) and three tests (pre, post, retention; *dataset_example_Mixed-design.sav*). The between-subject factor is stored in the dataset template variable “bs_1,” with the experimental group coded as 1 and the control group coded as 2. The values measured at each time of testing are stored in variables “ws_1” (pre-test), “ws_2” (post-test) and “ws_3” (retention-test), which together constitute the three levels of the within-subject factor.

**Figure 5:**
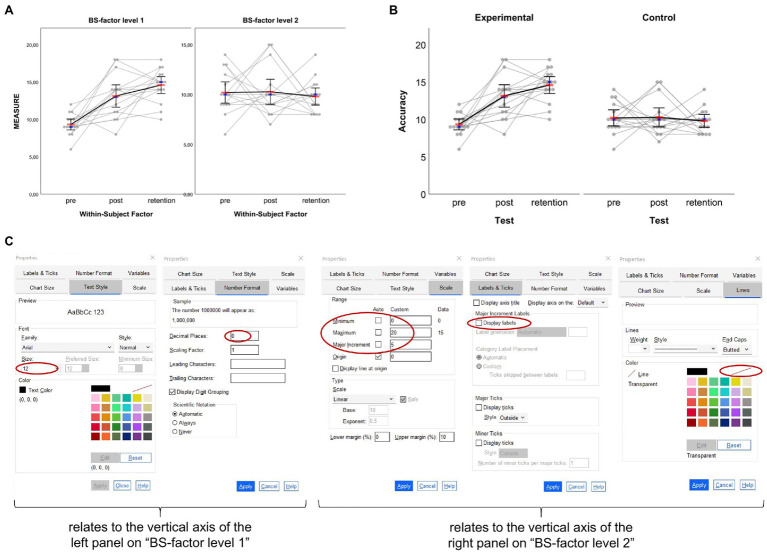
Line plots created based on the example dataset for two-factorial mixed-designs. **(A)** Original output based on syntax and **(B)** adjusted figure based on the settings illustrated and highlighted red in **(C)**. Please note that not each single step realized to move from **(A)** to **(B)** is shown in **(C)** (see main text for details). In **(A**,**B)** the red horizontal bars represent the arithmetic mean, the smaller blue horizontal bars represent the median and error bars represent 95% CIs associated with respective means.

The line plots shown in Figure 5A were created based on the syntax file *MIXED-Design_2-factors_2-x-3_LINE-plot__raw-dots-CONNECTED.sps* and the sixth visualization option provided therein to display arithmetic means (red bars) along with corresponding 95% CIs as well as respective medians (blue bars). As can be seen from Figure 5A, by default the graph is split into two panels according to the number of levels of the between-subject factor “bs_1” (three panels would be plotted if that factor had three levels). In each panel, data are shown as a function of the within-subject factor.

Importantly, unlike two-factorial between-subject design graphics, in mixed-design graphics properties of each panel’s vertical axis are independent from each other. To make this clear, numerical values are always shown for each panel’s vertical axis by default. This is important to keep in mind for further processing since depending on the underlying raw data distribution the range of values may differ between different groups, thus resulting in differently scaled vertical axes as is illustrated in Figure 5A. To arrive at the modified graphic shown in Figure 5B, where, among others, both panels’ vertical axes are aligned, the steps illustrated in Figure 5C were realized.

In a first step, properties that relate to the vertical axis with the largest range of values along that axis should be checked and fixed. In the example case, this means that properties of the left panel’s vertical axis (i.e., BS-factor level 1; “experimental” in the example) were checked (e.g., range of scale) and finalized (i.e., font size and number format; cf. Figure 5C). In a second step, properties of the right panel’s vertical axis (i.e., BS-factor level 2; “control” in the example) were adjusted to match with properties of the left panel’s vertical axis. In this regard, the most important step to make data shown in both panels comparable is to change settings for scale by using the values set for the first vertical axis (i.e., change minimum, maximum, and major increment). The remaining steps are rather cosmetic and thus optional. First, since both vertical axes are now numerically aligned, values may not necessarily be shown along the second vertical axis. To remove numerical labels on that particular axis, chose “Labels & Ticks” in the Properties Window and then uncheck “Display Labels.” Second, to make both panels look more coherent the vertical axis line of the second panel can be set invisible. To do so, chose “Lines” in the Properties Window and in the color section click on the color field that stands for full transparency (white field with red diagonal; see Figure 5C). In the example case, the latter step of setting axis line transparent was also applied to each panel’s top-horizontal axis (Figure 5B). Finally, here chart size was changed to a height of 10 cm with aspect ratio maintained (step not illustrated in Figure 5C) and axis titles as well as panel headings were adjusted to match with the fictitious study example.

In addition to the above, of course more than the aforementioned settings could have been applied to the graphic shown in Figure 5. For example, to make the blue bars representing median values larger and/or change their color, simply click on one of those markers, press CTRL + T to open the Properties Window and realize the settings needed. Note that this or other property changes would need to be done for each panel separately as both panels are defined independently from each other.

When reporting the results from studies like the former example, one may wish to not only visualize the raw (i.e., measured) data along with appropriate summary statistics as in Figure 5B but also visualize individual and summary performance changes between consecutive tests such as from pre- to post-test and from post- to retention-test (e.g., Weissgerber et al., 2016). The syntax collection allows for the creation of such additional visualization when the within-subject factor in mixed-designs has two or three levels (not supported for four or five levels). The corresponding syntax files are easy to spot in the collection since they include the term “with-Delta” in their name. Importantly, to create difference graphs along with the standard output dealt with before (Figure 5), no extra work on the dataset is needed. The syntax that allows the creation of difference graphs already includes code that does the work: it takes the values stored in the variables representing the different levels of the within-subject factor, calculates differences between consecutive conditions and stores difference values in variables 16 and 17 of the dataset template (see Figure 1). Note that, in the case of a three-levels within-subject factor, differences are only calculated between consecutive levels (i.e., level 2 minus level 1; level 3 minus level 2) and not between non-consecutive levels (i.e., not for level 3 minus level 1). Thus, calculation of those differences makes particularly (if not only) sense when the three levels of the within-subject factor follow a clear (e.g., temporal) order as is the case in the mixed-design training study example illustrated before. In such cases, I recommend to include a panel on raw difference scores in order to communicate individual differences between within-subject conditions more clearly (cf. Weissgerber et al., 2019).

**Figure 6:**
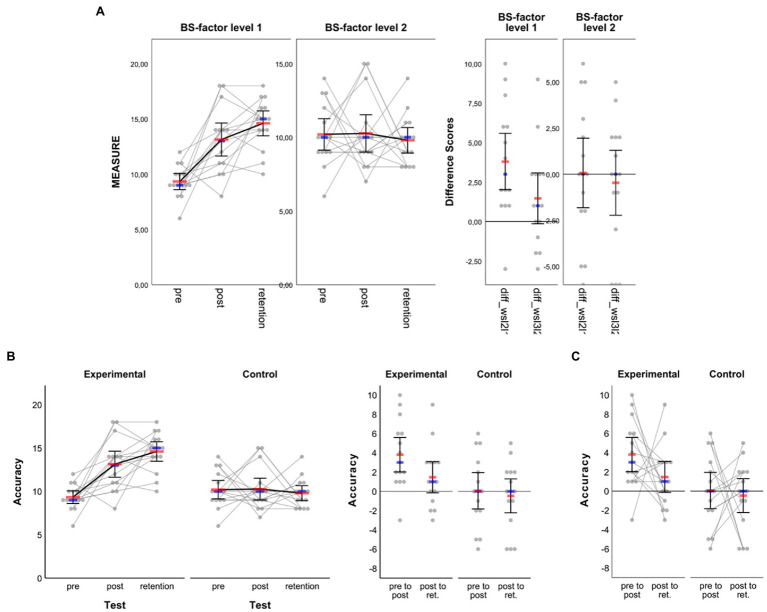
Line plots created based on the example dataset for two-factorial mixed-designs. In addition to what is shown in Figure 5, another panel is added which illustrates raw data and summary statistics for differences between consecutive within-subject factor levels (i.e., pre to post, post to retention). **(A)** Original output as obtained from syntax and **(B)** adjusted figure based on a variety of settings not illustrated here, but partially explained in Figure 5C as well as in the main text. **(C)** Panel with individual difference scores connected (see main text for details). In all panels, red horizontal bars represent arithmetic means, blue horizontal bars represent medians and error bars represent 95% CIs associated with respective means.

To illustrate the output of difference scores, the sixth visualization option from within the syntax file *MIXED-Design_2-factors_2-x-3_LINE-plot__raw-dots-CONNECTED__with-Delta-not-connected.sps* was applied to the same dataset as before, giving the output illustrated in Figure 6A. Admittedly, the output might appear of little use at first sight—especially the new panel on difference scores—but this is partly due to group-specific variance in the data and can be solved with only a few clicks. Having read up until this point and worked through the previous examples, readers know that the original graph’s properties can be changed easily to obtain a far nicer graph. To avoid unnecessary repetition and redundancy here, the steps realized to change the original output (Figure 6A) into the final output illustrated in Figure 6B are not highlighted in detail. Importantly, note that for the difference panel as well, data from both groups are treated independently so users would need to take care of the vertical axes being aligned numerically to make the groups’ difference scores look comparable. Of further note, as is illustrated in Figure 6C, one can also visualize intraindividual variation of the differences between adjacent within-subject factor levels by connecting the individual difference scores. This option of connecting individual difference scores is provided through syntax for mixed designs where the within-subject factor has three levels as in the current example. The corresponding syntax filenames end with “Delta-connected.” The syntax provided therein gives almost the same output as shown in Figures 6A,B with the only exception being that the individual difference scores in the right panel are connected as shown in Figure 6C. Visualizing intraindividual variation in the difference scores between adjacent within-subject factor levels can be an additional helpful means to communicate, for example, the (amount of) sustainability of pre-to-post changes through connection with post-to-retention changes (see also the discussion below in section Two-Factorial Within-Subject Design).

### Within-Subject Designs

Two example datasets are provided for pure within-subject designs, one to illustrate a one-factorial design with 4 levels (*dataset_example_WS-design_1-factor.sav*) and the other to illustrate a 2-x-2 design (*dataset_example_WS-design_2-factors.sav*). For the one-factorial design example, the dataset includes fictitious data from a study on the effects of acute exercise of varying intensities on choice reaction time closely inspired by Draper et al. (2010). The two-factorial design example, in turn, relates to a fictitious study inspired by Karageorghis et al. (2018) who investigated, among others, the combined effect of music tempi and intensities on hand grip strength.

#### One-Factorial Within-Subject Design

Since up until here the most relevant steps for adjusting an original graphical output in the SPSS Chart Editor have been described in detail, in this section the one-factorial within-subject design example is handled only briefly with focus on the different types of data visualization that can be created using the syntax collection, but without elaboration on the steps necessary to obtain the respective graphics. When visualizing data related to one-factorial within-subject designs, one first needs to decide on whether to connect the dots representing individuals’ raw data (syntax filenames include the string “raw-dots-CONNECTED”) or not (syntax filenames include the string “raw-dots-NOT-connected”). It is recommended to connect raw data dots only when the within-subject factor levels can be put in reasonable order as we might argue is the case in the fictitious example here, where the levels of the within-subject factor can be ordered according to increasing exercise intensity from rest to severe. Furthermore, as highlighted in the previous examples above, one can additionally choose between the type of visualization (i.e., box plot, dot plot or line plot) and within each of these types there is a variety of options on the measures one wishes to display in isolation or in combination (e.g., mean, median, SD, CIs; see Table 2).

**Figure 7:**
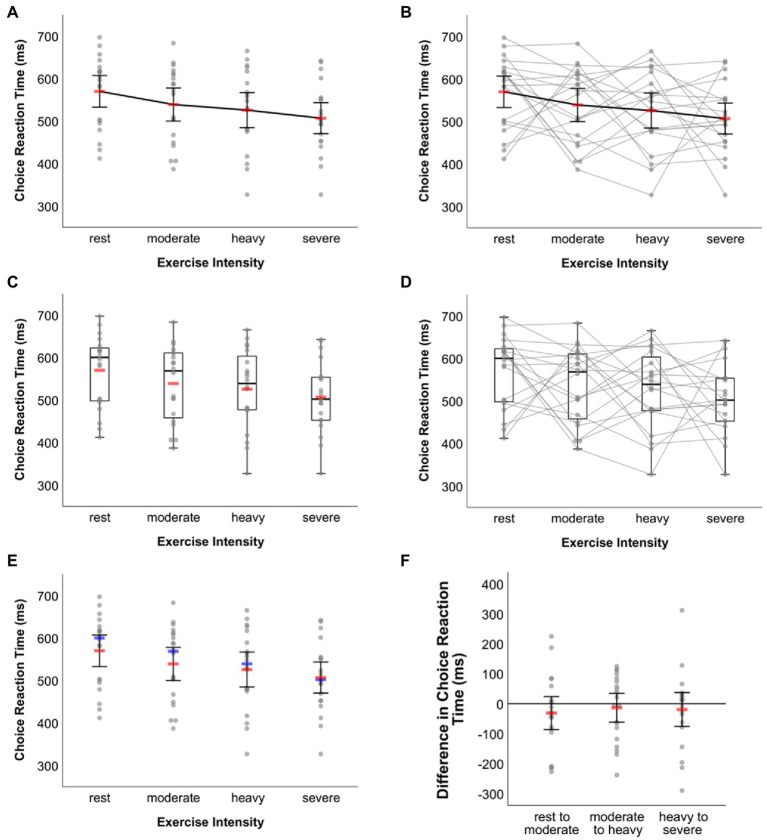
Exemplar plots visualizing the same data underlying the fictitious example for one-factorial within-subject designs. Line plot on mean choice RT **(A)** without and **(B)** with raw data connected. **(C)** Box plot together with mean choice RT without connection of raw data. **(D)** Box plot with raw data connected. **(E)** Dot plot together with mean and median choice RT without connection of raw data. **(F)** Dot plot of differences in choice reaction time between adjacent within-subject factor conditions (see main text for details). In **(A**,**B**,**E**,**F )** error bars represent 95% CIs associated with the respective means. Means are represented by red bars **(A–C**,**E**,**F )**, medians are indicated by blue bars **(E)**.

In the fictitious dataset, randomly generated data on choice reaction time measured in milliseconds under four exercise intensity levels are stored in variables “ws_1” (rest), “ws_2” (moderate), “ws_3” (heavy) and “ws_4” (severe). That is, the four factor levels are assigned to the SPSS variables in ascending order by intensity. Also, as can be inferred from the SPSS Variable View of the example dataset file, each variable is labelled according to the exercise intensity level it represents. Graphical outputs generated from the syntax templates maintain the order by placing the respective variables and data stored therein from left to right along a graph’s horizontal axis. The corresponding labels are also displayed along that axis. Figure 7 shows a selection of exemplar graphical outputs that were created by, first, running syntax from the following template files and, second, further adjustment in the SPSS Chart Editor as described along with the examples in the former sections:

- Figure 7A: syntax file *WS-Design_1-factor_4-levels_LINE-plot__raw-dots-NOT-connected.sps*, first visualization option therein: means & 95% CIs
- Figure 7B: syntax file *WS-Design_1-factor_4-levels_LINE-plot__raw-dots-CONNECTED.sps*, first visualization option therein: means & 95% CIs
- Figure 7C: syntax file *WS-Design_1-factor_4-levels_BOX-plot__raw-dots-NOT-connected.sps*, second visualization option therein: standard box plot & means (not connected)
- Figure 7D: syntax file *WS-Design_1-factor_4-levels_BOX-plot__raw-dots-CONNECTED.sps*, first visualization option therein: standard box plot
- Figure 7E: syntax file *WS-Design_1-factor_4-levels_DOT-plot.sps*; sixth visualization option therein: means, 95% CIs and medians

The zigzag pattern created through the grey lines connecting individual values in Figures 7B,D reveals the intra- and inter-individual variation in how different levels of exercise intensity affected choice reaction time. In this respect, these graphs are more informative than those given in Figures 7A,C,E where such information obviously cannot be inferred from. On the other hand, however, the zigzag pattern may make the respective graphs seem a little overloaded and thus difficult to read. Users wishing to visually communicate inter-individual variation of choice RT changes between adjacent exercise intensity conditions may then choose an alternative route. As explained above, the syntax-based creation of difference score visualizations is restricted to within-subject factors with up to three levels. In the present example, however, the factor has four levels. Still, this does not prevent from using syntax to display raw data along with summary statistics on these difference scores. All one has to do is to perform three little intermediate steps. In the example here, this would mean (i) to copy the original scores to new variables in the dataset (e.g., ws_1 [rest] copied to ws_1c, ws_2 [moderate] copied to ws_2c, etc.), (ii) to delete the original scores from variables ws_1 to ws_4, and (iii) to calculate difference scores between adjacent exercise intensity conditions and to store these scores in variables ws_1 (moderate [ws_2c]—rest [ws_1c]), ws_2 (heavy [ws_3c]—moderate [ws_2c]), and ws_3 (severe [ws_4c]—heavy [ws_3c]). Syntax for raw data visualization of one-factorial within-subject designs with three levels can now be applied to these three variables that include the difference scores. Further adjusting the output in the SPSS Chart Editor leads to Figure 7F (created from syntax file *WS-Design_1-factor_3-levels_DOT-plot.sps* and the first visualization option provided therein [*M* & 95% *CI*]).

#### Two-Factorial Within-Subject Design

Given the dependency of data within and across factors, raw data visualization gets a little more complicated in two-factorial within-subject designs. This could be one explanation for why recent calls and solutions for raw data visualization, to the best of my knowledge, did not elaborate on how to handle data originating from designs with more than one within-subject factor. Here, at least tentative solution for 2-x-2 and 2-x-3 within-subject designs is provided. The suggestion that follows considers that, for those designs, arithmetic means can easily be visualized as, e.g., line chart along with some measure of dispersion (i.e., CIs or SD) when running two-factorial repeated measures ANOVA in SPSS (“Analyze > General Linear Model > Repeated Measures…”). As an exception, given the dependency of data within and across factors, we may settle for not adding raw data to these outputs so as to also not challenge the readers’ eyes too much. Instead, since we are often interested in the differences between the conditions of within-subject factors, here the focus is on visualizing raw data on difference scores. Such outputs could be reported along with the puristic SPSS-RM-ANOVA outputs.

The syntax provided for two-factorial within-subject designs results in graphical outputs that include two panels: the first (i.e., left) panel shows individual data for the difference between adjacent conditions in factor B (two or three levels) separately for the two levels of factor A, whereas the second (i.e., right) panel shows individual data for the difference between levels 2 and 1 of factor A separately for the two or three levels of factor B. Similar to the designs covered in the previous sections, users can choose between different combinations of visualization of measures of central tendency (arithmetic mean and median) and dispersion (95% CI and SD). With regard to graph types, however, visualization is limited to dot plots with or without connection of individual difference scores (see Table 1).

**Figure 8:**
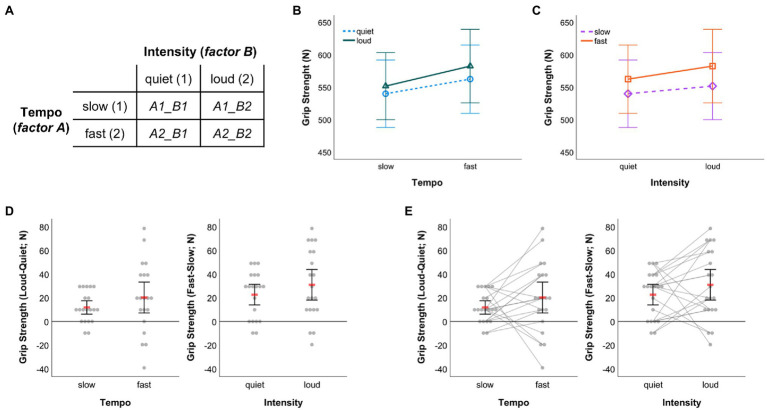
**(A)** Illustration of the exemplar 2-x-2 within-subject design. The labels of the cells representing factor level combinations correspond to the names of variables in the SPSS dataset template that need to be filled with values to run the data visualization syntax. Graphical outputs obtained from 2 × 2 RM-ANOVA showing arithmetic means and associated 95% CIs **(B)** for quiet and loud conditions by music tempo and **(C)** for slow and fast conditions by music intensity. **(D)** Dot plots visualizing individual differences between the loud and quiet condition under slow and fast tempo (left panel) as well as individual differences between the fast and slow condition under quiet and loud intensity (right panel). **(E)** Same as in **(D)** with individual data points additionally being connected through straight lines. In **(D**,**E)** red bars represent arithmetic means and error bars indicate 95% CIs associated with means.

In the following, fictitious data from a 2-x-2 within-subject design is used to illustrate data visualization options further (*dataset_example_WS-design_2-factors.sav*). To start with, the factorial design underlying the example is shown in Figure 8A. The four cells resulting from factor level combinations are labelled according to the names of the variables that need to be filled with measurement values in the SPSS dataset template to make the syntax work. Importantly, put in the values that were originally measured. The difference scores that are to be displayed will be calculated automatically when running syntax code.

When running a 2 × 2 RM-ANOVA on the data, figures like the ones shown in Figures 8B,C can be created from within SPSS (RM-ANOVA main menu > “Plots…” > assign one factor to the horizontal axis and create separate lines by the other factor, choose line chart as chart type and include 95% CIs as error bars). The graphical outputs resulting from that procedure were further edited using the SPSS Chart Editor to obtain the versions displayed in Figures 8B,C. The outputs illustrated in Figures 8D,E were created with code from the syntax collection (*WS-Design_2-factors_2-x-2_DOT-plot__raw-dots-NOT-connected__Delta-only.sps* and *WS-Design_2-factors_2-x-2_DOT-plot__raw-dots-CONNECTED__Delta-only.sps*; first visualization option [*M* & 95% CI] provided therein) and both outputs were processed further using the SPSS Chart Editor as described in the sections above. In both of these panels, individual difference scores between conditions of one factor (e.g., intensity) are depicted separately for each level of the other factor (e.g., tempo) along with the arithmetic mean and 95% CI.

While the same data are shown in Figures 8D,E, the key difference is that individual difference scores are connected in Figure 8E. The latter has the advantage that one can additionally communicate in how far the effect of one factor (e.g., intensity) was consistent across conditions in the other factor (e.g., tempo) across individuals. For example, from pure visual inspection of the direction of gray lines depicted in the left panel of Figure 8E, one can infer that six participants showed larger grip strength difference in favor of the loud intensity condition under slow as opposed to fast tempo (four of them even demonstrated a reversal towards larger grip strength under quiet than loud intensity when tempo was fast), while the remaining 14 participants demonstrated larger grip strength difference, to varying degree, in favor of the loud intensity condition under fast compared to slow tempo. Such information obviously is not available when individual scores are not connected (Figure 8D). However, it can be visualized easily using syntax and be referred to during an in-depth discussion of study findings, potential practical implications and perspectives for future research (Nimphius and Jordan, 2020). Therefore, my recommendation would be to almost always opt for visualizing the connection of individual data points. That strategy might only not be feasible when the number of participants whose data are to be visualized exceeds a value of about 20 (Teare, 2016) to 30 (Fosang and Colbran, 2015) such that the resulting graph might become overloaded and impossible to read.

## Data Visualization Beyond the Designs Explicitly Covered by Syntax

The SPSS syntax collection focuses on visualizing univariate data related to standard one- and two-factorial research designs. However, the application of syntax is not limited to the designs described in the previous sections. Instead, with some creativity the syntax templates can even be used beyond the scenarios illustrated. More generally, if one has a multi-factorial design but wishes to visualize selected main effects or two-way interactions for which the application criteria for syntax are met (see section The SPSS Syntax Collection; Figure 1), one can create the required raw data visualization with the syntax and dataset template provided.

To exemplify further, assume a study with an underlying three-factorial mixed-design that includes two between-subject factors, with at least one of these factors having no more than three levels, and one within-subject factor with a maximum of five levels. Further assume that one wishes to visualize the three-way interaction. Putting all data belonging to that interaction into one graph makes it difficult to read irrespective of whether raw data is displayed or not. Since raw data should be displayed, one could do the following: Visualize the two-way interaction between one between-subject factor (store that factor in variable “bs_1”; note that this factor may not have more than 3 levels) and the within-subject factor (measurements stored in variables “ws_1,” “ws_2,” …). Further, store values that relate to the second between-subject factor in variable “bs_2.” Now, the solution is that the syntax for data visualization is run separately for each level of the second between-subject factor stored in “bs_2.” To do so, in SPSS choose “Data > Select Cases… > If condition is satisfied….” In the window that appears, type in “bs_2 = 1” (without quotation marks) to de-activate all cases in the dataset that do not satisfy that condition. Then, confirm all settings, switch to the syntax file that contains relevant code and run the code snippet to create the favorite graph (*cf.* section Two-Factorial Mixed-Designs). Now, the two-way mixed interaction is displayed for level 1 of the second between-subject factor. Next, change the selected cases by changing the corresponding command to “bs_2 = 2,” which de-activates all cases in the dataset that do not satisfy that particular condition. Again, run the same visualization syntax as before to create the two-way mixed interaction display for level 2 of the second between-subject factor. If that factor has more than two levels, repeat the previous steps for level 3 and so on. When finished with the visualization of selected cases contingent on the second between-subject factor, one can further modify the individual graphs according to the steps described in section Example Applications. Importantly, the visualization syntax does not work but would result in error messages if the dataset file was split according to the variable “bs_2.”

As another example, let us assume that the two-factorial 2 × 2 within-subject design example illustrated in section Two-Factorial Within-Subject Design were extended to a three-factorial mixed-design where the effects of music tempo and intensity on hand grip strength were tested in two independent groups of athletes such as judoka and climbers. Similar to the example some lines above, the syntax collection can be used to visualize the two-way within-subject interaction separately for both groups of athletes. To do so, store values representing group membership (i.e., judoka or climber) in the grouping variable “bs_1,” select cases for one particular group (e.g., judoka) and run syntax code, then select cases for the other group (e.g., climbers) and run the same syntax code again. This leads to visualizations of individual difference scores for both groups. More simple plots illustrating only the arithmetic means along with, e.g., CIs could be prompted through the “Repeated Measures…” procedure provided in SPSS along with the computation of inferential statistics.

With the above examples, I hope it has become clear that with some creativity one can visualize data for even more designs than those explicitly covered through syntax (cf. Table 1) and solutions similar to the ones above might apply to other study designs as well.

## Modification of Syntax

The SPSS syntax collection includes pre-specified settings that some users may wish to modify before running code. This would simply require replacing selected code snippets with the code one wants to have included in the customized syntax. Table 3 gives an exemplar overview of syntax code that might be interesting to modify such as the level for CIs (pre-set to 95%), the display of standard error of the mean instead of SD, changing graphic settings related to, for example, color, shape, and size or the arrangements of dots representing raw data (by default arranged symmetrically with central alignment relative to the central position of a category without superimposing individual dots). For in-depth information on syntax for creating graphs using SPSS, interested readers are recommended to consult the GPL Reference Guide for IBM SPSS Statistics (IBM, 2016) and related publications (Wilkinson, 2005; McCormick et al., 2017).

**Table 3:** Overview of exemplar aspects users might want to modify in syntax.

Aspect to modify	Default syntax code	Comment for modification
Level of CI	alpha(0.95)	Change value in parenthesis to, e.g., 0.90 or 0.99 to visualize 90% or 99% CIs. By default, the value is set to 0.95 in all syntax templates that allow the visualization of 95% CIs.
Display of SEM instead of SD	region.spread.sd	Change “sd” to “se” in the code such that the modified code reads: region.spread.se
Shape	shape(shape.ibeam) *[used for dispersion measures]* shape(shape.solid) *[used for straight lines]*	The possibility of changing the shape of a graphical element may depend on its exact type such as “interval” to display dispersion measures (i.e., CI, SD), “point” to display raw data values or measures of central tendency (i.e., mean, median) or “line” to connect values (raw data dots or means). To change a graphic element’s shape in syntax, change the code given in parenthesis after “shape.” Since there is a multitude of options available for shape, please see the “GPL Reference Guide for IBM SPSS Statistics” (IBM, 2016) for details.
Color	color.interior(color.grey) color.exterior(color.grey)	To change the color of, for example, the dots representing raw data, simply change the color code written in parenthesis to, e.g., red, green, blue, black or any other of the many color constants available through GPL.^1^
Size	size(size.medium) size(size.“6px”)	The size of graphic elements is either indicated through size constants (e.g., medium in the left column) or through explicit values given in pixels (e.g., “6px” in the left column). To change an element’s size by modifying code, simply replace the code given in parenthesis after “size.” either through another constant (tiny, small, medium, large, huge) or through another value (e.g., “8px”, “10px”).
Transparency	transparency.interior(transparency.“0.4”) transparency.exterior(transparency.“0.4”)	Transparency values can range between 0 (no transparency) to 1 (full transparency). Change value in parenthesis (default is 0.4) to change transparency so as to put more or less emphasis on raw data dots compared to measures of central tendency and dispersion (Weissgerber et al., 2019).^1^
Arrangement of dots representing raw data	point.dodge.symmetric	Change “symmetric” to “asymmetric” for asymmetric arrangement of raw data dots with left alignment relative to the central position of a category (no overlap of dots representing the same value).

1 The code snippet “interior” relates to a graphic element’s fill, whereas “exterior” relates to its border. If no such specification is made (i.e., neither interior nor exterior) code is implicitly handled as with “interior.”

## Conclusion

Inspired by the paucity of practical solutions available for transparent data visualization using IBM SPSS Statistics, here a free-to-use collection of more than 100 syntax files was introduced to encourage and to facilitate users of SPSS to create transparent visualizations of summary statistics and its underlying raw data. The collection and the tutorial provided with this article hopefully add a valuable piece to the growing possibilities for the transparent visualization of quantitative data. Importantly, apart from some basic understanding of how SPSS works, the creation of visualizations does not require any programming skills or the like. Consequently, the syntax collection hopefully is of value for an extended target group in various fields of research and teaching. While the examples used in this article originate from the domain of sport psychology, of course the syntax is not restricted to that particular area but can similarly be applied to continuous quantitative data from any other field in psychology and beyond.

The syntax collection’s scope of application focusses on common one- and two-factorial study designs, but can also be used to visualize data from even higher-factorial designs. The visualization of raw data as illustrated here is particularly suitable when the number of cases included per group or experimental condition is not too high (i.e., about 20 to 30 cases; Fosang and Colbran, 2015; Teare, 2016), especially when the dots representing individual raw data are connected through lines. Otherwise, if there is a large number of cases to visualize at least the connection of dots may render a graphic quite useless as the individual connections may be impossible to identify. Instead, for (very) large datasets other forms of visualization are recommended such as the creation of violin plots (Weissgerber et al., 2019) or raincloud plots (Allen et al., 2021) to visualize distributions. Such plots can be created for common one- or two-factorial study designs, for example, using free software packages like JASP (JASP Team, 2022) or JAMOVI (The Jamovi Project, 2021). Of note for those interested in immersing themselves in SPSS syntax programming, creating violin-like plots is not impossible in SPSS as is illustrated, for instance, in a blog post by Wheeler (2012) and an online video tutorial (how2stats, 2019; a SPSS syntax file applicable to a one-factorial between-subject design is provided alongside the video). Additional integration of such graph type in this article’s accompanying syntax collection, however, is beyond its intended scope.

It needs to be acknowledged that a number of excellent, however often not for free graphic programs (*R* is a notable exception) are available that allow raw data visualizations like the ones presented here as well (e.g., Origin, GraphPad PRISM, and Matlab). Compared to these programs, the possibilities offered by SPSS for extensive raw data visualization are limited. Still, even users of SPSS can get more out of their continuous quantitative data than they might have thought of before. In this respect, the present work adds to the various technical solutions for raw data visualization that are available by now.

When visually inspecting the outputs generated with the syntax collection, it needs to be kept in mind that the CIs plotted around arithmetic means are unadjusted stand-alone CIs (Cousineau et al., 2021). These intervals are of limited use for the comparison between groups and particularly problematic with regard to comparing between factor levels in within-subject designs (Loftus and Masson, 1994). Users interested in visualizing adjusted CIs are recommended to consider the *R*-based library superb (Cousineau et al., 2021).

The graph types for which SPSS syntax files are provided here were selected based on recent recommendations by Weissgerber et al. (2019), assuming that the types cover large part of the visualization options required to make univariate raw data resulting from common factorial designs transparent. As a potential limitation, usability of the syntax files and the creation of figures based thereon was tested only in a non-systematic way by asking colleagues for feedback, but not by running a large-scale user study which, however, also was not within the scope of the present work.

As another limitation, the visualization options discussed herein relate to univariate data only. In view of the increasing richness and complexity of data structures obtained from, for example, sensors (e.g., psychophysiological measures), audio and video recordings, appropriate data visualization for effective combination of multimodal data and communication of essential scientific results becomes even more a challenge. In this respect, more sophisticated visualization tools such as SubjectBook (Taamneh et al., 2016),^5^ ChronoViz (Fouse et al., 2011)^6^ and ELAN (Wittenburg et al., 2006)^7^ may turn out helpful.

In conclusion, the present syntax collection and tutorial are meant to facilitate univariate raw data visualization for those who work with SPSS. Neither visualization tool, however, compensates or even substitutes the logical and oftentimes challenging exercise of developing a sound theoretical and methodological basis for a research question that one aims to preliminarily answer on the basis of quantitative data. Once that exercise has been completed and data have been collected successfully, users of SPSS may find the syntax collection accompanying this article helpful for making their raw data transparent.

## Data Availability Statement

The original contributions presented in the study are included in the article/Supplementary Material, and further inquiries can be directed to the corresponding author.

## Author Contributions

The author confirms being the sole contributor of this work and has approved it for publication.

## Conflict of Interest

The author declares that the research was conducted in the absence of any commercial or financial relationships that could be construed as a potential conflict of interest.

## Publisher’s Note

All claims expressed in this article are solely those of the authors and do not necessarily represent those of their affiliated organizations, or those of the publisher, the editors and the reviewers. Any product that may be evaluated in this article, or claim that may be made by its manufacturer, is not guaranteed or endorsed by the publisher.

## Footnotes

^1^ http://statistika.mfub.bg.ac.rs/interactive-linegraph/

^2^ https://rawgraphs.io/

^3^ https://www.estimationstats.com

^4^ https://huygens.science.uva.nl/PlotsOfData/

^5^ http://subjectbook.times.uh.edu/

^6^ https://chronoviz.com/

^7^ https://archive.mpi.nl/tla/elan
